# Human Prune Regulates the Metabolism of Mammalian Inorganic Polyphosphate and Bioenergetics

**DOI:** 10.3390/ijms241813859

**Published:** 2023-09-08

**Authors:** Ernest R. Scoma, Renata T. Da Costa, Ho Hang Leung, Pedro Urquiza, Mariona Guitart-Mampel, Vedangi Hambardikar, Lindsey M. Riggs, Ching-On Wong, Maria E. Solesio

**Affiliations:** 1 Department of Biology, and Center for Computational and Integrative Biology, Rutgers University, Camden, NJ 08103, USA; 2 Department of Biological Sciences, Rutgers University, Newark, NJ 07102, USA; andy.leung@sahmri.com (H.H.L.);

**Keywords:** inorganic polyphosphate (polyP), h-Prune, polyP’s metabolism, mammalian bioenergetics

## Abstract

Inorganic polyphosphate (polyP) is an evolutionarily conserved and ubiquitous polymer that is present in all studied organisms. PolyP consists of orthophosphates (Pi) linked together by phosphoanhydride bonds. The metabolism of polyP still remains poorly understood in higher eukaryotes. Currently, only F_0_F_1_-ATP synthase, Nudt3, and Prune have been proposed to be involved in this metabolism, although their exact roles and regulation in the context of polyP biology have not been fully elucidated. In the case of Prune, in vitro studies have shown that it exhibits exopolyphosphatase activity on very short-chain polyP (up to four units of Pi), in addition to its known cAMP phosphodiesterase (PDE) activity. Here, we expand upon studies regarding the effects of human Prune (h-Prune) on polyP metabolism. Our data show that recombinant h-Prune is unable to hydrolyze short (13–33 Pi) and medium (45–160 Pi) chains of polyP, which are the most common chain lengths of the polymer in mammalian cells. Moreover, we found that the knockdown of h-Prune (h-Prune KD) results in significantly decreased levels of polyP in HEK293 cells. Likewise, a reduction in the levels of polyP is also observed in *Drosophila melanogaster* loss-of-function mutants of the h-Prune ortholog. Furthermore, while the activity of ATP synthase, and the levels of ATP, are decreased in h-Prune KD HEK293 cells, the expression of ATP5A, which is a main component of the catalytic subunit of ATP synthase, is upregulated in the same cells, likely as a compensatory mechanism. Our results also show that the effects of h-Prune on mitochondrial bioenergetics are not a result of a loss of mitochondrial membrane potential or of significant changes in mitochondrial biomass. Overall, our work corroborates the role of polyP in mitochondrial bioenergetics. It also demonstrates a conserved effect of h-Prune on the metabolism of short- and medium-chain polyP (which are the predominant chain lengths found in mammalian cells). The effects of Prune in polyP are most likely exerted via the regulation of the activity of ATP synthase. Our findings pave the way for modifying the levels of polyP in mammalian cells, which could have pharmacological implications in many diseases where dysregulated bioenergetics has been demonstrated.

## 1. Introduction

Inorganic polyphosphate (polyP) is a ubiquitous polymer that is highly conserved through evolution and is present in all studied organisms [1,2]. Structurally, it consists of a chain of orthophosphates (Pi) linked together by phosphoanhydride bonds, identical to those present in ATP [2,3]. In mammalian cells, polyP has not been extensively studied, mostly due to the lack of reliable, comprehensive, and specific models and methods. However, it has been proposed that, in these cells, polyP is present in the micromolar range, and its chain length spans from three to several hundred residues long [4,5,6]. PolyP can be found in various intracellular locations in mammalian cells, including the cytoplasm, the nucleus, and even in the extracellular space [4,7]. However, we and others have shown a high presence of polyP in mitochondria [8,9,10,11]. The molecular structure of polyP and its high abundance in mitochondria make this polymer an excellent candidate to be involved in the regulation of energy production within the organelle. In fact, different researchers have demonstrated that polyP is involved in cellular bioenergetics at different levels, including the regulation of mitochondrial calcium homeostasis, and the formation and opening of the mitochondrial permeability transition pore [3,9,12,13,14,15]. However, the exact mechanism by which polyP exerts these effects remains to be fully understood, even though the direct regulation of the balance between glycolysis and mitochondrial oxidative phosphorylation (OXPHOS) [8], and the regulatory effects of Nudt3-Zn^+2^ in polyP [16], have been described as part of this mechanism.

The metabolism of polyP in bacteria and yeast is well understood. For example, in yeast, the eukaryotic vacuolar transporter chaperone complex (VTC) is responsible for the synthesis of polyP [17,18]. Moreover, in bacteria, two polyP kinases have been described. These enzymes are polyphosphate kinase 1 (PPK1), which catalyzes the reversible transfer of the terminal γ-phosphate of ATP to polyP; and polyphosphatase kinase 2 (PPK2), which can synthesize polyP from both ATP and GTP [19,20,21]. In the same organisms, the enzymes responsible for the hydrolysis of polyP are exopolyphosphatase (PPX, which releases terminal Pi from the polyP chain), and endopolyphosphatase (PPN, which releases internal Pi) [22,23,24]. However, mammalian homologs for these enzymes have not yet been found. Accordingly, the enzymes responsible for the metabolism of polyP in mammalian cells still remain far from being totally understood, despite recent advances. Specifically, it has been proposed that both F_0_F_1_-ATP synthase and the phosphohydrolase Nudt3 could be involved in this process [16,25]. However, the exact regulation of these enzymes in the context of polyP biology remains unclear, and other proteins could also be involved in the metabolism of the polymer. While F_0_F_1_-ATP synthase is a classical mitochondrial enzyme, Nudt3 is mainly localized in the nucleus [26]. These diverse locations open the door to questions regarding whether the pools of polyP in the mammalian cell could be different depending on where the polymer is synthesized, as well as whether polyP is able to pass membranes, and if so, how this transport occurs.

Another protein that has been suggested to play a role in the metabolism of polyP in higher eukaryotes is Prune [27,28]. Prune is a member of the desert hedgehog homolog (DHH) family of phosphoesterases, which also includes bacterial and yeast cytosolic PPX1 [29]. Prune exhibits short-chain exopolyphosphatase activity in vitro on three and four Pi-long polyP [27]. Such activity was reported to be four orders of magnitude greater than its cAMP phosphodiesterase (PDE) activity [27]. It should be noted, however, that the common consensus among the polyP research community is that polyP is formed of more than three or four Pi, although the exact number might change in the different tissues [4,6,30,31]. The orthologue of human Prune (h-Prune) in *Drosophila melanogaster* (*Drosophila*), Prune, localizes to mitochondria (in human cells, this protein is mostly found in the cytoplasm and nucleus, but it can also be present in other parts of the cells), where it downregulates cAMP signaling to stabilize TFAM, which is a mitochondrial transcription factor that promotes mitochondrial DNA (mtDNA) replication [28]. While the role of h-Prune in human cancer progression has already been described [32], little is known about the activity of h-Prune in the mammalian metabolism of polyP.

Here, we expand our knowledge regarding the role of Prune in the metabolism of human polyP. Specifically, we show that h-Prune is not able to hydrolyze short- and medium-chain polyP (13–33 and 45–160 Pi, respectively). In fact, our results show that when h-Prune is knocked down, the levels of polyP decrease in HEK293 cells. These findings indicate that h-Prune is required to maintain proper levels of polyP, rather than solely participating in the catabolism of the polymer. The same effect is observed in *Drosophila*, which demonstrates the conserved function of Prune in the metabolism of polyP in other eukaryotes. Our findings, together with those in the available literature, suggest a chain-specific effect of Prune on the metabolism of the polymer. Moreover, the loss of h-Prune inhibits the activity of ATP synthase, and it decreases the production of ATP, via a mechanism that is independent of the regulation of the mitochondrial membrane potential or the mitochondrial content. Probably as a consequence of these effects on polyP metabolism, knocking down h-Prune in mammalian cells has a powerful effect on mitochondrial bioenergetics. Overall, our results show the sharp regulatory effects of Prune on polyP metabolism and, therefore, on mitochondrial bioenergetics. They suggest that these effects are mediated by the modulation of ATP synthase activity. Therefore, the regulation of h-Prune activity could be a valid and powerful pharmacological target in human pathologies where mitochondrial bioenergetics is affected, such as neurodegenerative disorders [33].

## 2. Results

### 2.1. Recombinant h-Prune Is Able to Release 5′AMP from cAMP, but It Does Not Hydrolyze Medium- and Long-Chain PolyP

**Figure 1:**
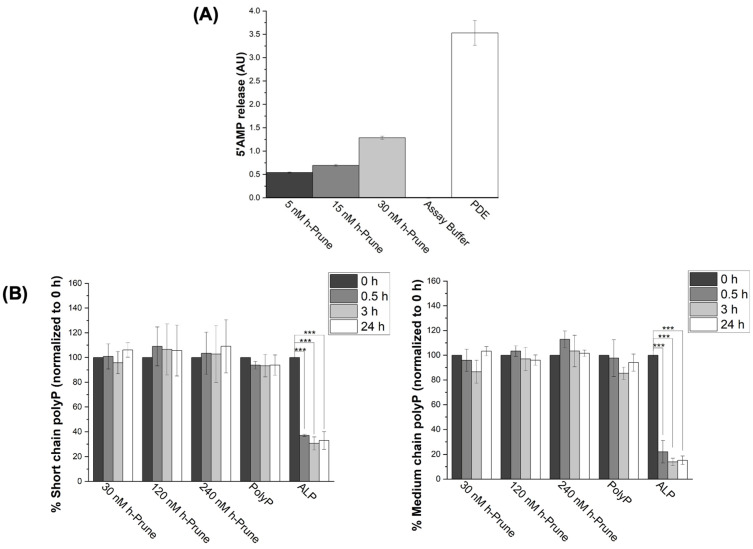
Recombinant h-Prune does not hydrolyze short (13–33 Pi)- and medium (45–160)-chain polyP, even though it conserves its ability to release 5′AMP from cAMP. (**A**) Graph showing that recombinant h-Prune conserves its PDE activity. To conduct this in vitro study, we assayed the release of 5′AMP from cAMP. Increasing concentrations of h-Prune with 10 mM MgCl_2_ were tested. PDE was used as a positive control, and assay buffer as a negative control. (**B**) h-Prune is not able to hydrolyze short (13–33 Pi) and medium (45–160 Pi) chains of polyP after 24 h of the assay in the presence of 5 mM MgCl_2_. These chain lengths are those that should be more common in mammalian cells. The levels of polyP were assayed using DAPI fluorescence, and ALP was used as positive control. The results are expressed as mean ± SEM. The PDE assay (as it is an enzymatic study) was conducted using experimental triplicates, while the other experiments were conducted using at least experimental and biological triplicates. *** *p* ≤ 0.001.

By measuring the release of 5′AMP from cAMP, we assayed the PDE activity of recombinant h-Prune, in the presence of increasing concentrations of h-Prune. Our data show a concentration-dependent increase in the levels of 5′AMP after a 30 min incubation with the enzyme, as we increased the levels of h-Prune (2.3-fold increase when comparing the levels of 5′AMP in the presence of 5 nM h-Prune to those in the presence of 30 nM h-Prune). The assay buffer did not show any phosphodiesterase activity, while the PDE enzyme was used as a positive control for these experiments (Figure 1A). Subsequently, we assayed the plausible PPX activity of recombinant h-Prune, using increasing concentrations of short- (13–33 Pi) and medium-chain (45–160 Pi) synthetic polyP. A total of 5 mM Mg^2+^ was added as a cofactor. As previously described, mammalian polyP is proposed to be in this range of lengths [4,6,30,31]. Our data show that h-Prune was unable to hydrolyze polyP under these experimental conditions. Alkaline phosphatase (ALP) was used as a positive control for these experiments (Figure 1B).

### 2.2. PolyP Levels Are Significantly Decreased in h-Prune-Knockdown (KD) HEK293 Cells, and This Effect Is Conserved in Drosophila

**Figure 2:**
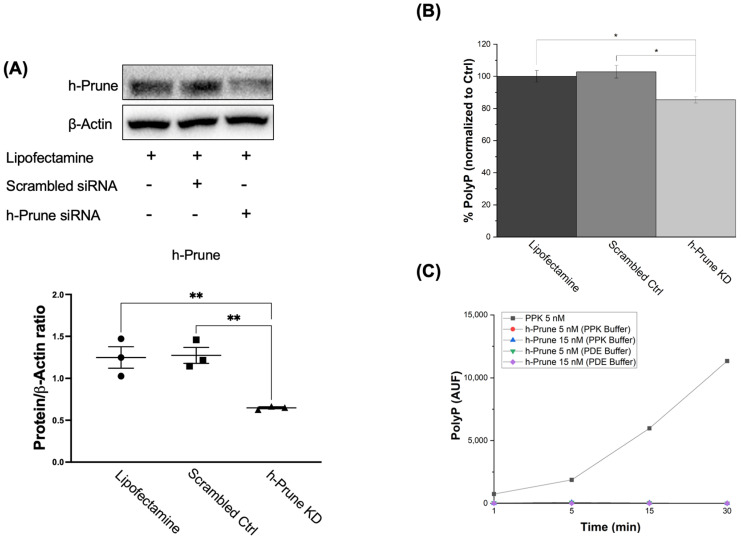
h-Prune KD HEK293 cells show decreased levels of polyP. (**A**) Significant immunoblots and densitometric analysis that demonstrate decreased levels of h-Prune in HEK293 cells after transfection with siRNA. Note that transfection with scrambled siRNA does not induce any significant changes in the levels of h-Prune. In the densiometric analysis, circles represent individual values from samples treated with lipofectamine; and squares and triangles individual values of samples transfected with Scrambled siRNA and h-Prune siRNA, respectively. (**B**) Graph showing the levels of polyP in our cellular models. This quantification was conducted by measuring the fluorescence of DAPI-polyP. The levels of polyP are decreased by knocking down Prune. Transfection with the scrambled siRNA did not induce any significant effects on the levels of polyP. (**C**) The plausible kinase activity of h-Prune was also assayed. However, no variations in the levels of polyP were found in this case either. PPK was used as a positive control, and h-Prune was incubated either in the PPK or the PDE buffer. The results are shown as mean ± SEM. The PPK assay (as it is an enzymatic study) was conducted using experimental triplicates, while the other experiments were conducted using at least experimental and biological triplicates. * *p* ≤ 0.05, ** *p* ≤ 0.01.

Transfection with h-Prune siRNA decreased the levels of h-Prune in our samples by 48% (*p* value = 0.005). Transfection with scrambled siRNA, and treatment with lipofectamine, did not induce any effects on these levels (Figure 2A). Once we set up our experimental conditions, to determine the effects of h-Prune on the intracellular levels of polyP, we assayed the presence of the polymer in our samples by measuring the DAPI-polyP fluorescence [34]. Our data show that h-Prune KD HEK293 cells have significatively decreased levels of polyP (Figure 2B). Interestingly, h-Prune did not show any direct kinase activity (Figure 2C).

**Figure 3:**
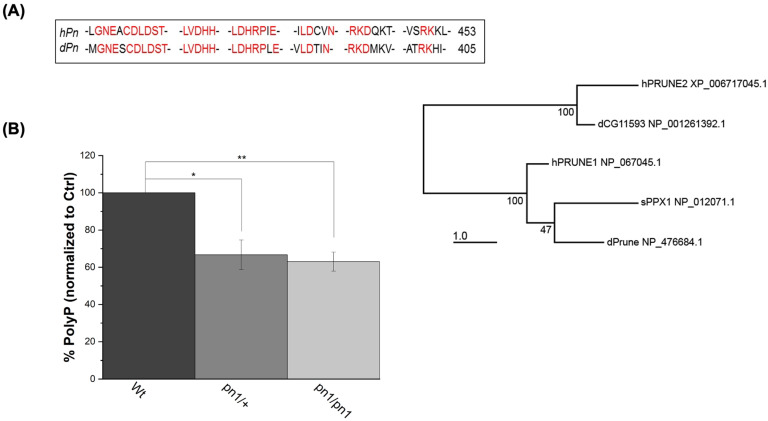
The effects of h-Prune KD on the levels of polyP are conserved between humans and *Drosophila.* (**A**) Phylogenetic analysis of the protein sequences shows that the Prune orthologs are highly conserved between humans and *Drosophila*. This is especially significant in the PPX consensus sequence, which is marked in red in the sequence alignment. The numbers on the right are the residues on the remaining C-terminal fragment. (**B**) Assay of the levels of polyP (using DAPI-polyP fluorescence) in homozygous (*pn^1^*/*pn^1^*) and heterozygous mutants. Results are shown as mean ± SEM of at least three independent experiments. * *p* ≤ 0.05, ** *p* ≤ 0.01.

To address whether the effects observed in h-Prune KD HEK293 cells are also present in other species, we decided to investigate the conserved ortholog in *Drosophila prune (pn)*. The phylogenetic analysis reveals that the *Drosophila* prune protein is closely related to PRUNE1, which encodes for h-Prune [35] (Figure 3A). The protein sequence alignment of the N terminal PPX consensus sequences of *Drosophila* Prune and h-Prune indicates a strong homology between these two proteins, including an abundance of invariant residues within these aligned sequences (Figure 3A). Subsequently, we extracted and quantified the levels of polyP in the Wt control (*Canton S*; *CS*) and *Prune* mutant (*pn^1^*) *Drosophila* heads. Also, in these samples, we found that the loss of one copy of Prune was sufficient to cause a significant reduction in the levels of polyP, which resulted in comparable levels to those found in homozygous mutants (39% decrease in homozygosis—*p* value = 0.009—vs. 37% decrease in heterozygosis—*p* value = 0.026), (Figure 3B). Thus, similarly to what is observed for h-Prune, *Drosophila* Prune is also required for the maintenance of the levels of polyP. Our findings demonstrate that Prune orthologs have an evolutionarily conserved function in the regulation of the homeostasis of polyP.

### 2.3. ATP Levels Are Decreased in h-Prune KD HEK293 Cells: This Effect Is Mediated by a Drop in the Activity of Mitochondrial ATP Synthase

**Figure 4:**
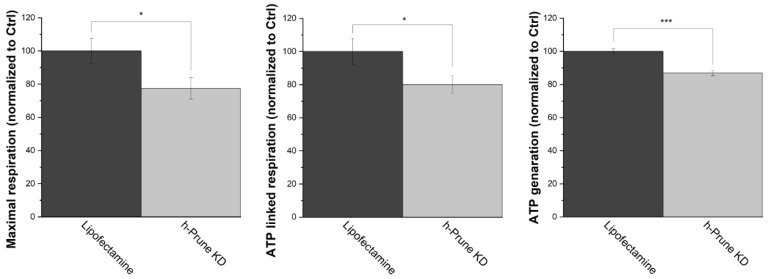
h-Prune KD deleteriously affects mitochondrial respiration, decreasing the cellular levels of ATP. Using Oroboros 2k respirometry, ATP-linked respiration (**left**) and maximal respiration (**center**) were assayed in HEK293 cells. Our data show that these two parameters are decreased in h-Prune KD, compared to the control samples. Moreover, the cellular levels of ATP (**right**), which were analyzed using luminescence, are also decreased when h-Prune is knocked down. The results are shown as mean ± SEM of at least three independent experiments. * *p* ≤ 0.05, *** *p* ≤ 0.001.

Mammalian F_0_F_1_-ATP synthase has been proposed as one of the enzymes involved in the metabolism of polyP in these organisms [25]. However, the exact regulation of this enzyme in the context of the metabolism of polyP remains mostly unknown. To address the plausible regulatory effects of h-Prune in F_0_F_1_-ATP synthase, as well as the consequences of decreased levels of polyP on bioenergetics, we decided to first assay the production of ATP in our samples, using biochemical tools and Oroboros 2k respirometry. Our data show a significant drop in both ATP-linked respiration (20%, *p* value = 0.043) and the maximal respiration (23%, *p* value = 0.027) in h-Prune KD HEK293 cells. Consequently, the total levels of cellular ATP are also decreased in these samples (13%, *p* value = 4.93 × 10^−4^) (Figure 4).

**Figure 5:**
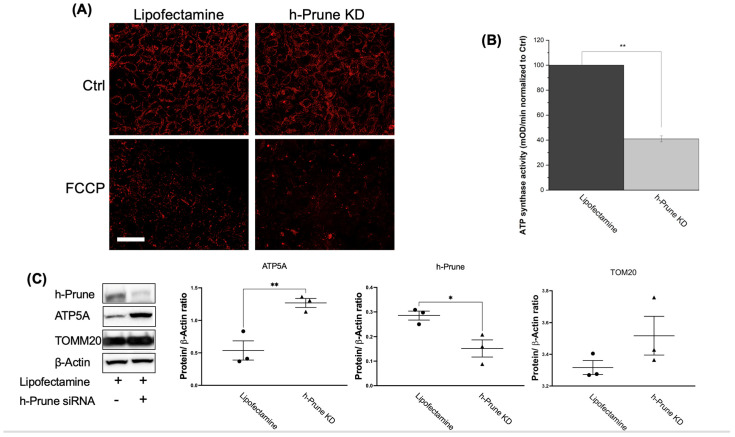
In HEK293 cells, the effects of h-Prune on the levels of polyP are mediated by the regulation of the activity of ATP synthase, but not by an effect on the expression of ATP synthase, or on the regulation of mitochondrial membrane potential or mitochondrial biomass. (**A**) Significant images obtained after loading our cells with TMRM. Note that no major differences are found between control and h-Prune KD HEK293 cells. FCCP 10 μM was used as a positive control to induce mitochondrial depolarization. (**B**) Assay of the activity of ATP synthase in our samples. Note that decreased levels of the activity of this enzyme are found in h-Prune KD, compared to the levels shown in the control samples. (**C**) Significant immunoblots and densitometric analysis that corroborate that h-Prune KD HEK293 cells have significantly decreased levels of the protein. Moreover, using the same methods, our data show that the levels of ATP5 are increased in h-Prune KD, while those of TOM20 are not affected by knocking down Prune. In the densiometric analysis, circles represent individual values from samples treated with lipofectamine, while triangles represent individual values from h-Prune KD samples. The results are shown as mean ± SEM of at least three independent experiments. Scale bar: 50 μM. * *p* ≤ 0.05, ** *p* ≤ 0.01.

Since mitochondrial membrane potential is crucial to producing ATP, we decided to investigate whether the loss of h-Prune impacts this potential. To conduct these studies, we used TMRM staining. Our results show that both control and h-Prune KD HEK293 cells exhibit similar levels of TMRM fluorescence, in both resting and depolarized (after treatment with FCCP) conditions (Figure 5A). Subsequently, we studied the activity of ATP synthase in control and KD HEK293 cells. While the activity of this enzyme is significantly decreased in h-Prune KD (59%, *p* value = 7.64 × 10^−5^) (Figure 5B), the levels of the protein assayed via immunoblotting are significantly increased in the same samples (54%, *p* value = 0.006) (Figure 5C). This increase is not a consequence of a rise in the number of mitochondria, as no significant differences were observed between the control and h-Prune KD in the levels of TOMM20 (Figure 5C). Together, these data indicate that h-Prune regulates the activity of ATP synthase, which has been proposed to be involved in the production of polyP [25].

## 3. Materials and Methods

### 3.1. Reagents

Dulbecco’s Modified Eagle medium (DMEM), penicillin–streptomycin, Hank’s Balanced Salt Solution (HBSS), tris(hydroxymethyl)-1,3-propanediol hydrochloride (TRIS-HCl), and trypsin were purchased from Gibco-Invitrogen (Carlsbad, CA, USA). Carbonyl cyanide-4 (trifluoromethoxy) phenylhydrazone (FCCP), antimycin A, poly-L-lysine, Tween-20, tetramethylrhodamine methyl ester perchlorate (TMRM), ammonium sulfate, ethylenediaminetetraacetic acid (EDTA), dimethyl sulfoxide (DMSO), methanol, phenol, creatine phosphate, creatine kinase, chloroform, magnesium chloride salt, sodium chloride salt, potassium chloride salt, heat-inactivated fetal bovine serum (FBS), agar, Brewer’s yeast, cornmeal, propionic acid, and Tegosept were purchased from Sigma-Aldrich (St. Louis, MI, USA). 40-6-diamino-2-phenylindole (DAPI), phosphate-buffered saline (PBS), HEPES-KOH (pH 7.5), triton X-100, lipofectamine, alkaline phosphatase (ALP), Pierce Halt protease and phosphatase inhibitor cocktails, Pierce BCA protein assay kit, and Pierce ECL Western blotting substrate were purchased from Thermo Fisher Scientific (Waltham, MA, USA). Trypan blue, and all the materials and reagents used in the immunoblotting experiments were obtained from BioRad (Hercules, CA, USA). ATP luminescent measurement kits and recombinant h-Prune (ab153466) were purchased from Abcam (Cambridge, Cambridgeshire, UK); PolyP standards were a gift from Dr. Toshikazu Shiba (Kitasato University, Tokyo, Japan).

### 3.2. Cell Culture

HEK293 cells were obtained from the American Type Culture Collection (ATCC, Manassas, VA, USA). Cultures were maintained in accordance with ATCC guidelines, and following our previously published protocols [9,12]. Briefly, cells were thawed and grown in DMEM supplemented with 10% (*v*/*v*) FBS and 1% penicillin/streptomycin (5000 U/mL, and 5000 µg/mL, respectively), and placed in an incubator at 37 °C and 5% CO_2_.

### 3.3. Transfections

We seeded 2.5 × 10^5^ cells per well in 6-well plates. After 48 h, transfections were conducted using h-Prune siRNA (sc-75218, Santa Cruz Biotechnology, Dallas, TX, USA), following the manufacturer’s protocol. Briefly, for each well, 60 pmol siRNA was complexed with 6 μL of lipofectamine for 20 min in serum-free DMEM. Cells were then washed twice with serum-free medium before the transfection mixture was added. Subsequently, cells were maintained in the incubator for 5 h in this mixture. Controls were incubated with either scrambled siRNA (sc-37007 Santa Cruz Biotechnology, Dallas, TX, USA) or solely lipofectamine. Transfection medium was then replaced with regular culture medium and cells were left in the incubator for 24 h before harvesting.

### 3.4. Immunoblotting Assays

Immunoblots were performed as previously described [13,36]. β-actin was used as a loading control. Primary antibodies were used at a 1:1000 dilution unless otherwise stated. The following antibodies were used for immunoblotting: anti-h-Prune (anti-mouse, sc-393318, Santa Cruz Biotechnology, Dallas, TX, USA), anti-TOMM20 (anti-rabbit, CST42406S, Cell Signaling Technology, Danvers, MA, USA), anti-ATP5A (anti-mouse, ab110273, Abcam, Cambridgeshire, UK), and anti-β-Actin (anti-mouse, ab8226, Abcam, Cambridgeshire, UK). Anti-rabbit and anti-mouse secondary antibodies (1706516 and 1706515, respectively) were purchased from BioRad (Hercules, CA, USA) and used at a 1:2000 dilution. Densitometric analysis was performed using ImageJ software 1.53t (NIH, Bethesda, MD, USA).

### 3.5. Quantification of Cellular PolyP

Samples for polyP quantification were washed and scraped on ice with cold PBS, and centrifuged at 200× *g* for 10 min at 4 °C. Cell pellets were resuspended in 25 μL of lysis buffer (30 mM Tris-HCl, pH 7.4, 200 mM KCl, 0.5% Triton, 1× protease inhibitor) and spun on a rotator for 30 min at 4 °C. Samples were then sonicated on ice at 30% amplitude for 3 cycles with 15 s on and 30 s off, and subsequently centrifuged at 20,000× *g* for 10 min at 4 °C. Supernatants were then collected and protein concentrations were determined via BCA assay. Samples were then brought to a final protein concentration of 0.1 µg/µL in a buffer containing 50 mM Tris HCl, pH 7.4. A total of 50 μL of each sample was added to each well of a black 96-well plate in triplicate. Samples were then incubated for 25 min with 10 μM DAPI in the dark, at room temperature. Fluorescence intensity was measured at 415/550 nm, using a ClarioStar multifunctional microplate reader (BMG LABTECH, Ortenberg, Germany).

### 3.6. PolyP Measurement of Drosophila

Forty 1-week-old flies were snap-frozen in liquid nitrogen and decapitated via vortexing. Fly heads were collected using a pre-chilled double-layered sieve with different mesh sizes (0.6 mm and 0.4 mm). Subsequently, fly heads were homogenized with a pellet mixer in 100 μL lysis buffer containing 0.5% Triton X-100, 5 mM EDTA, and 1x protease inhibitor in PBS. After centrifugation at 12,000× *g* for 10 min at 4 °C, the supernatant was pipetted into a 0.5 mL microcentrifuge tube and stored at −80 °C. PolyP was organically extracted from these samples, following a neutral phenol/chloroform and ethanol precipitation protocol, which was previously described in [37], and modified in [38]. The obtained polyP pellets were resuspended in 50 μL of 50 mM Tris Hcl, pH 7.4. A total of 1 μL of each sample was loaded into 99 μL of the same buffer in triplicate, in a well from a black 96-well plate. Samples were then incubated with 10 μM DAPI for 25 min. Fluorescence intensity was measured at 415/550 nm using a ClarioStar multifunctional microplate reader (BMG LABTECH, Ortenberg, Germany).

### 3.7. h-Prune PDE and PPX Activity Assay

The PDE activity of h-Prune (used at different concentrations) was assayed after 30 min using a cyclic nucleotide PDE assay kit (BML-AK800-0001, Enzo Life Sciences, Farmingdale, NY, USA), following the manufacturer’s protocol, and as previously described [39,40]. The PPX activity of h-Prune was assayed by measuring the degradation of polyP, as previously described by our group [14]. Briefly, 5 mM of synthetic polyP of different chain lengths (short and medium polyP) were incubated in the presence of 5 mM MgCl_2_ and increasing concentrations of h-Prune (30 nM, 120 nM, and 240 nM), and 20 μM DAPI. Subsequently, DAPI-polyP fluorescence was quantified at different time points (0 h, 0.5 h, 1 h, 3 h, and 24 h) using a ClarioStar multifunctional microplate reader (BMG Labtech, Ortenberg, Germany). A total of 1 U of alkaline phosphatase/well was added as a positive control to induce the degradation of polyP. Assays were conducted at pH = 7.4.

### 3.8. PPK Activity Assay

PPK activity assays were conducted following a previously published protocol [41]. The same concentrations of h-Prune as those used in the other activity assays were also used in this experiment. Assays were conducted either in a solution containing 50 mM HEPES-KOH (pH 7.5), 50 mM ammonium sulfate, 5 mM MgCl_2_, 20 mM creatine phosphate, and 60 μg mL^−1^ of creatine kinase, or in h-Prune PDE assay buffer (BML-KI181-0040, Enzo Life Sciences, Farmingdale, NY, USA) supplemented with 10 mM MgCl_2._ Reaction mixtures were prewarmed to 37 °C and the reaction was started by adding MgATP. PPK isolated from *Escherichia coli*, which was used as positive control in these experiments, was a gift from Dr. Gray’s laboratory (University of Alabama at Birmingham, Birmingham, AL, USA). Assays were conducted at pH = 7.4.

### 3.9. Assay of Cellular ATP Levels

After transfection with h-Prune siRNA and protein quantification using BCA and following the manufacturer’s protocol, cellular levels of ATP were assayed using the Luminescent ATP Detection Kit (ab113849, AbCam, Cambridge, UK), also in this case following the manufacturer’s protocol and as we previously described [12]. Luminescence was assayed using a ClarioStar multifunctional microplate reader (BMG Labtech, Ortenberg, Germany).

### 3.10. Oroboros Assays

Mitochondrial respiratory capacity was determined via high-resolution respirometry using an Oroboros O2K Oxygraph (Oroboros Instruments, Innsbruck, Austria) following a slightly adjusted version of the protocol previously described in [42]. Briefly, 7.5 × 10^5^ cells were seeded in 75 cm^2^ flasks, transfected following the protocol previously described in this paper, and incubated for 24 h under regular growth conditions. Cellular respiration was then assessed at 37 °C, while the samples were stirred at 750 rpm in Mir05 medium (Oroboros Instruments, Innsbruck, Austria). To assess different respiratory states, 0.1 μM oligomycin, 0.5 μM CCCP, and 0.05 μM antimycin A were added to the cells as the oxygen consumption rate (OCR) was measured. Using this protocol, we were able to determine ATP-linked respiration, and maximal respiration. The OCR was recorded using DataLab 4 software (Oroboros Instruments); it is expressed as pmol of O_2_/s per 10^6^ cells, and it was normalized by the number of cells. All the respiratory states were subsequently corrected for non-mitochondrial respiration (ROX).

### 3.11. ATP Synthase Activity Assay

ATP synthase activity was assayed using the ATP synthase Enzyme Activity Microplate Assay Kit (ab109714, AbCam, Cambridge, UK), and following the manufacturer’s protocol. This method is based on the measurement of absorbance at 340 nm. Reduction of this absorbance is a consequence of the oxidation of NADH to NAD^+^, a process that is coupled with the conversion of ATP to ADP by ATP synthase. Buffer blanks were used as negative controls and 5 µM Oligomycin A was used as a positive control to block the activity of the enzyme.

### 3.12. TMRM Assay

We seeded 10 × 10^5^ cells in poly-L-Lysine-treated coverslips. After 48 h, cells were either untreated or transfected according to the previously described protocol. Samples were then incubated in HBSS containing 20 nM TMRM for 20 min at 37 °C in the dark. Subsequently, the medium was replaced with HBSS containing 5 nM, and cells were imaged using confocal microscopy (LSM8, Leica Microsystems, Wetzlar, Germany), following the same protocol that we used before, including the addition of 10 μM FCCP as a positive control for mitochondrial depolarization at the end of the experiment.

### 3.13. Drosophila Husbandry

Flies were grown in vials containing standard fly food (3 L of food contained 25.6 g agar, 80.6 g brewer’s yeast, 190.6 g cornmeal, 40.3 g sugar, 16.6 g propionic acid, and 13.3 g Tegosept) [43]. Fly vials were kept in an incubator set at 25 °C with a 12 h/12 h light/dark cycle. *Drosophila Prune* mutant *Pn^1^* was obtained from Bloomington Drosophila Stock Center (stock number 174), and it was previously described [44]. Wild-type (Wt) strain *Canto-S (SC)* was a gift from others and has been used in previous studies [43,45].

### 3.14. Phylogenetic Analysis

CLUSTALW (Kyoto University Bioinformatics Center, www.genome.jp/tools/clustalw/, accessed on 28 August 2023) was used to perform protein sequence alignment, and to construct a phylogram. Bootstrap tests (100) were performed using PhyLM [46]. Bootstrap values are given at the branch nodes.

### 3.15. Statistical Analysis

All data are presented as mean ± SEM. The specific number of samples that were used in each experiment is stated in the figure legends. Statistical assays were conducted using Students’ *t*-test and Tukey post hoc two-way ANOVA. Statistical significance levels were set at α = 0.05 (* *p* ≤ 0.05, ** *p* ≤ 0.01, and *** *p* ≤ 0.001). Statistical analysis was performed and graphical representations created using OriginLab 10.05 and GraphPad Prism 10.0.2 (Northampton, MA, USA and San Diego, CA, USA, respectively).

## 4. Discussion

The important role played by polyP in the maintenance of mammalian physiology, including bioenergetics, has already been shown by us and others [3,8,9,12,13,14,15,16,47]. This regulatory role in energy production and storage is conserved throughout evolution [48,49,50]. Interestingly, dysregulated levels of polyP have been proposed to be involved in the etiopathology of different human diseases, including some neurodegenerative and hematological disorders [51,52,53]. However, our current knowledge of the functions and the mechanisms in charge of the regulation of polyP, especially in mammalian organisms, is still scarce. This is mostly due to the limited literature available regarding the metabolism of the polymer, despite the recent findings in which Prune, mitochondrial F_0_F_1_-ATP synthase, and Nudt3 have been proposed to be involved in this process [16,25,27].

Prune is a protein that has been well conserved through evolution, and is involved in metastasis and tumor expansion in humans [32]. This protein is able to hydrolyze up to four Pi-long chains of polyP [27]. However, our data show that recombinant h-Prune cannot hydrolyze short- and medium-chain polyP (13–33 and 45–160 Pi, respectively) after 24 h, and that Prune does not exhibit exopolyphosphatase activity in either HEK293 mammalian cells or *Drosophila*, in our working conditions. The differences between our findings and those previously described could be a consequence of the specific length of the polyP used in the experiments. In fact, it has already been shown that h-Prune exhibits exopolyphosphatase activity on very short chains of polyP [27]. However, in this work, we used significantly longer chains of polyP in our studies. Longer chains (13–33 and 45–160) seem to align better with the expected length of polyP in mammalian cells [4,5,6]. Based on our results and the literature, Prune could contribute to both the synthesis and the hydrolysis of polyP, depending on the length of the polymer. Length-dependent effects of the enzymes involved in the metabolism of polyP have already been demonstrated. For example, endopolyphosphatase 1 from *Saccharomyces cerevisiae* (the homolog of this enzyme in mammalian cells remains unknown) exhibits exo- and endo-polyphosphatase activities, depending on the length of polyP that is present at each moment [54]. Moreover, the exopolyphosphatase activity of Prune could be dependent on the levels and the availability of some specific binding partners. In fact, it has been demonstrated that the binding of Prune to nm23-h1, a metastasis suppressor gene, negatively affects the exopolyphosphatase activity of the protein [55]. Other factors that could influence the exopolyphosphatase activity of Prune could be the specific composition of ions [27], as well as the plausible location of the protein in different subcellular locations, such as mitochondria.

Our findings show not only that Prune is not primarily an exopolyphosphatase under our working conditions, but also that Prune is involved in the regulation of the synthesis of polyP in both HEK293 and *Drosophila*. This effect is conserved between the two species, which could be explained by the ancient nature of the polymer, and by the fact that the sequences of the genes that code for h-Prune and *Drosophila* Prune exhibit high similarity, particularly in the PPX consensus sequence. Interestingly, in the case of *Drosophila*, our data show no significant differences in the levels of polyP between the heterozygous and the homozygous KD. This could suggest that the total lack of polyP is incompatible with life in these organisms, and therefore, some minimal levels of the polymer need to be maintained. In fact, polyP has been found in every studied organism [56], which supports the idea that it is a crucial polymer for living organisms. Null *Drosophila* alleles were not used because the color of the eyes, produced as a consequence of the mutation, interferes with the DAPI-polyP fluorescence.

The levels of polyP and the status of the electron transfer chain, which is crucial to maintaining the proper mitochondrial membrane potential, are intimately related [57]. Therefore, the decreased levels of polyP observed in h-Prune KD could be a consequence of a direct effect of Prune on this potential. However, our data rule out this possibility, as no major differences were observed when cells were labeled with TMRM. Due to the close relationship between mitochondria and the metabolism of polyP [8,9,15,25,57], another possibility could be that knocking down h-Prune could directly affect mitochondrial content in our HEK293 cells, which could, in turn, decrease the levels of ATP synthase or of other mitochondrial enzymes that could potentially be involved in the metabolism of polyP. However, our findings show no significant differences in the levels of TOMM20, which is a well-studied and conserved protein and a component of the outer mitochondrial membrane [58], between the control and the h-Prune KD samples.

In fact, the effects of Prune in the metabolism of polyP seem to be mediated by the regulation of the activity of ATP synthase, an enzyme that has been proposed to be involved in the metabolism of polyP [25]. In support of this possibility, when h-Prune is knocked down in our HEK293 cells, OXPHOS-related ATP production, and therefore, the total levels of ATP are decreased, which could be a consequence of the Prune-induced decreased activity and/or the presence of ATP synthase. Decreased OXPHOS is also observed in MitoPPX cells, that is, cells enzymatically depleted of mitochondrial polyP [14]. To further investigate this, we assayed both the activity and the protein levels of ATP synthase in our samples. Our data show a significant decrease in the activity of ATP synthase, assessed by measuring the activity of ATP5A, which is a main component of the catalytic subunit of F_0_F_1_-ATP synthase [59], in h-Prune KD HEK293 cells. However, the levels of this protein were significantly increased in the same samples, which could suggest a compensatory mechanism to maintain cell viability and some minimal energetic production. In fact, decreased activity of ATP5A has been described in pathologies where dysregulated bioenergetics and increased apoptosis are present, such as Alzheimer’s Disease [33,60]. Further studies aimed at clearly identifying the effects of purified Prune on the activity and regulation of ATP synthase in vitro will help to elucidate the exact relationship between Prune and this enzyme.

Overall, our findings show that Prune regulates the levels of polyP in both mammalian cells and *Drosophila*. Specifically, decreased expression of h-Prune induces a drop in the levels of polyP. The effects of Prune on the metabolism of polyP are probably exerted via the regulation of ATP synthase, and this affects the levels of cellular ATP, either by decreasing the activity of ATP synthase, or by affecting the equilibrium between the levels of polyP and those of ATP (the direct interconversion between polyP and ATP has already been shown [61]). Our results contribute to increasing our still highly limited knowledge regarding the metabolism of polyP, and they establish the modulation of Prune as a valid strategy to modify the levels of the polymer in mammalian cells. As mentioned above, dysregulated levels of polyP have been found in models of different human diseases [51,52,53].

## Data Availability

Further information and requests for resources and reagents should be directed to and will be fulfilled by the Lead Contact, Maria E. Solesio (m.solesio@rutgers.edu).
